# Bamboo‐Inspired Crack‐Face Bridging Fiber Reinforced Composites Simultaneously Attain High Strength and Toughness

**DOI:** 10.1002/advs.202308070

**Published:** 2023-12-28

**Authors:** Hao Wang, Zhangyu Wu, Jie Tao, Bin Wang, Chaobin He

**Affiliations:** ^1^ Department of Materials Science and Engineering National University of Singapore Queenstone 117575 Singapore; ^2^ Department of Mechanical Engineering City University of Hong Kong Hong Kong 999077 China; ^3^ School of Materials Science and Engineering Southeast University Nanjing 210096 China; ^4^ School of Materials Science and Technology Nanjing University of Aeronautics and Astronautics Nanjing 210096 China; ^5^ Institute of Materials Research and Engineering Agency for Science Technology and Research (A*STAR) Fusionopolis Way Innovis 138634 Singapore

**Keywords:** bamboo‐inspired structure design, carbon material reinforced composite, face‐bridging fiber reinforced composite, simultaneously strong and tough

## Abstract

Biological strong and tough materials have been providing original structural designs for developing bioinspired high‐performance composites. However, new synergistic strengthening and toughening mechanisms from bioinspired structures remain yet to be explored and employed to upgrade current carbon material reinforced polymer composites, which are keystone to various modern industries. In this work, from bamboo, the featured cell face‐bridging fibers, are abstracted and embedded in a cellular network structure, and develop an epoxy resin/carbon composite featuring biomimetic architecture through a fabrication approach integrating freeze casting, carbonization, and resin infusion with carbon fibers (CFs) and carbon nanotubes (CNTs). Results show that this bamboo‐inspired crack‐face bridging fiber reinforced composite simultaneously possesses a high strength (430.8 MPa) and an impressive toughness (8.3 MPa m^1/2^), which surpass those of most resin‐based nanocomposites reported in the literature. Experiments and multiscale simulation models reveal novel synergistic strengthening and toughening mechanisms arising from the 2D faces that bridge the CFs: sustaining and transferring loads to enhance the overall load‐bearing ability and furthermore, incorporating CNTs pullout that resembles the intrinsic toughening at the molecular to nanoscale and strain delocalization, crack branching, and crack deflection as the extrinsic toughening at the microscale. These constitute a new effective and efficient strategy to develop simultaneously strong and tough composites through abstracting and implenting novel bioinspired structures, which contributes to addressing the long‐standingly challenging attainment of both high strength and toughness for advanced structural materials.

## Graphical abstract

The distinct bamboo structure of cell crack‐face bridging fibers embedded in a cellular network is identified, which is essential to the remarkable mechanical properties, and this structure is implemented by making a cellular carbon nanotubes‐containing network that bridges CFs via cell faces to create new composites that attain both outstanding strength and toughness.

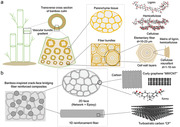

## 1 Introduction

**Figure 1:**
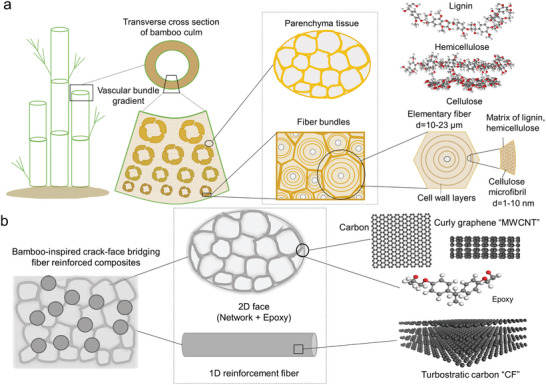
Bamboo‐inspired crack‐face bridging fiber reinforced composites. a) Hierarchical structure of bamboo. The bamboo culm can be considered as vascular bundles (fiber bundles) embedded in a matrix of cellular parenchyma tissue; each fiber bundle consists of aligned elementary fibers (10–23 µm in diameter) that are composed of concentric cell‐wall layers, in which cellulose microfibrils (about 1–10 nm in diameter) are embedded in a matrix rich in hemicellulose and lignin. b) Inspired by the cell crack‐face bridging fiber bundles embedded in a cellular network structure of bamboo, crack‐face bridging carbon fiber reinforced composites are designed. The CFs resemble the fiber bundles in bamboo which are bridged by 2D faces containing carbon and multi‐walled carbon nanotubes (MWCNTs) that form a cellular network surrounding the CFs. The CFs are formed by turbostratic carbon while MWCNTs by curved graphene. Such face‐bridging CFs in a cellular network are infused with resin, forming the bamboo‐inspired crack‐face bridging fiber reinforced composite material.

Attaining both high strength and toughness with lightness has been a challenging pursuit for advanced structural materials, in which bioinspired material design has made exciting research progress.^[^ ^1^, ^2^, ^3^, ^4^, ^5^, ^6^, ^7^, ^8^ ^]^ Specifically, bamboo is composed of solely organic constituents but is among the toughest and strongest biological materials^[^ ^9^ ^]^ with a high strength‐to‐weight ratio, due to a unique hierarchical structure (**Figure** **1**a).^[^ ^1^, ^10^, ^11^ ^]^ Bamboo consists of fiber bundles embedded in a matrix of cellular parenchyma tissue, with each fiber bundle formed by aligned elementary fibers (about 10–23 µm in diameter^[^ ^11^ ^]^). Each elementary fiber have concentric cell‐wall layers that are composed of nanoscale stiff cellulose microfibrils embedded in a hemicellulose/ligin matrix.^[^ ^1^, ^11^ ^]^ Despite extensive research studying bamboo structure and mechanical properties on the remarkable specific strength and toughness, the distinct cellular microstructure featuring solid, stiff fibers^[^ ^12^ ^]^ embedded in a porous matrix that provides abundant cell faces to bridge the fibers, which gives rise to the structural efficiency for superior mechanical performance,^[^ ^1^ ^]^ has not been explored in detail.

In comparison, engineering carbon material reinforced polymer composites have been widely used in aviation/aerospace, marine, and automobile industries, mostly due to their high stiffness, strength, and lightweight,^[^ ^13^ ^]^ while the frequently low ductility and poor fracture toughness hinder giving a full play to these materials.^[^ ^14^ ^]^ The reinforcing carbon nanomaterials, which include carbon nanotubes (CNTs), graphene, and carbon fibers (CFs) that have been considered as turbostratic carbon sheets possess high strength and modulus and lightweight structure^[^ ^15^, ^16^, ^17^ ^]^; while the extensively used thermoset polymers as matrices (e.g., epoxies)^[^ ^13^ ^]^ also show brittle mechanical properties. Numerous existing research on improving the toughness of carbon material reinforced composites focus largely on chemical manipulations over composition^[^ ^18^ ^]^ and interfaces^[^ ^19^ ^]^ with different degrees in success (Tables s2 and S3, Supporting Information), yet employing effective structural toughening mechanisms from biological tough and strong materials through appropriate fabrication techniques into carbon material reinforced composites remains to be developed.

In this work, we abstract the bamboo‐inspired structure of cell crack‐face bridging fibers that reinforce a cellular network; we then implement such structure by integrating CNTs to obtain the cellular network which bridge the CFs via cell faces to create high‐performance composites with both outstanding strength and toughness (Figure 1b). Taking into consideration the efficacy of freeze casting method in assembling building blocks to obtain bioinspired hierarchical structures for superior mechanical properties,^[^ ^20^, ^21^, ^22^, ^23^ ^]^ we create bamboo‐inspired crack‐face bridging fiber reinforced composites by utilizing freeze casting‐assisted assembly of nanoscale CNTs and microscale CFs to mimic the symbolic structure of bamboo (Figure 1). The CFs resemble the fiber bundles in the bamboo, and the cellular network containing CNTs and show 2D cell faces bridging CFs are the counterpart of the cellular parenchyma tissue in bamboo. The cellular network is carbonized and further infused with resin, yielding the bamboo‐inspired crack‐face bridging fiber reinforced composite material.

## 2 Results and Discussion

### 2.1 Fabrication and Characterization of the Bamboo‐Inspired Crack‐Face Bridging Fiber‐Reinforced Composites (BFFs)

**Figure 2:**
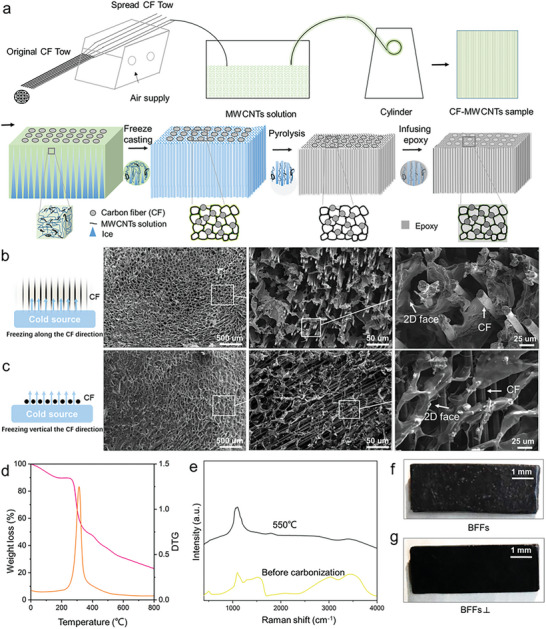
Fabrication and characterization of bamboo‐inspired crack‐face bridging fiber reinforced composites (BFFs). a) Fabrication process: CFs are dispersed and incorporated into a chitosan solution containing MWCNTs. Subsequently, freeze casting and drying are performed, followed by carbonization, and resin infiltration. Scanning electron microscopy (SEM) images of the 2D face‐bridging fibers embedded by the cellular network after carbonization: b) the freezing direction parallel to the CF, c) the freezing direction perpendicular to the CF. d) Thermo‐gravimetric analysis (TGA) is conducted to examine the network after freeze‐drying. e) The Raman spectroscopic analysis of the CF‐bridging cellular network. Photographs of the BFFs: f) BFFs (freezing direction parallel to the CF), g) BFFs⊥ (freezing direction perpendicular to the CF).

The fabrication process of bamboo‐inspired crack‐face bridging fiber reinforced composites (BFFs) is depicted in Figure 1 and **Figure** **2**a. In this process, CF bundles are initially dispersed in a solution containing chitosan (CS) and multi‐walled CNTs (MWCNTs) and then subjected to freeze casting and drying. The freeze drying step helps to create a cellular structure holding the CFs together with desired alignment through cell faces connecting CFs, which mimicks the featured bamboo microstructure of directional fibers embedded in the cellular parechyma. After this, a carbonization treatment is performed to modify the faces of the cellular network; this converts the organic components into carbon, resulting in a carbonized cellular network containing MWCNTs. Subsequently, this system of face‐bridging CFs embedded in a cellular network is infused with epoxy resin, creating a solid, bamboo‐inspired face‐bridging fiber reinforced composite material. The composite materials with the bridging faces parallel to the CFs (fabricated by controling the freezing direction parallel to the CFs) are referred to as BFFs, while BFFs⊥ denote those with the bridging faces perpendicular to the CFs.

The microstructures of the carbonized cellular network with cell faces bridging CFs are shown in Figure 2b,c. The aligned growth of ice crystals induced by temperature gradients leads to the formation of a cellular network containing MWCNTs and bridging the CFs. When the freezing direction aligns with the axis of the CFs, the formed microscale cellular network show cell faces parallel to the CFs to bridge the CFs (Figure 2b). Likewise, when the freezing direction is perpendicular to the CF axis, the cell faces of the cellular network are perpendicular to the CFs (Figure 2c).Thermogravimetric analysis results indicate that the CF‐bridging cellular network exhibits significant weight loss at 330°C and reaches a stable mass after 530°C (Figure 2d). From the Raman spectra of this cellular network in Figure 2e, the prominent bands near 1250 cm^−1^ can be attributed to disordered carbon and graphitic carbon.^[^ ^24^ ^]^ After carbonization at 550°C in N_2_, the CF‐bridging cellular network consists mainly of carbon (Figure 2e). Figure 2f,g shows the bamboo‐inspired face‐bridging fiber reinforced composites after epoxy infusion with the bridging cell faces parallel (BFFs) and perpendicular (BFFs⊥) to the CFs.

### 2.2 The Mechanical Properties of the BFFs

**Figure 3:**
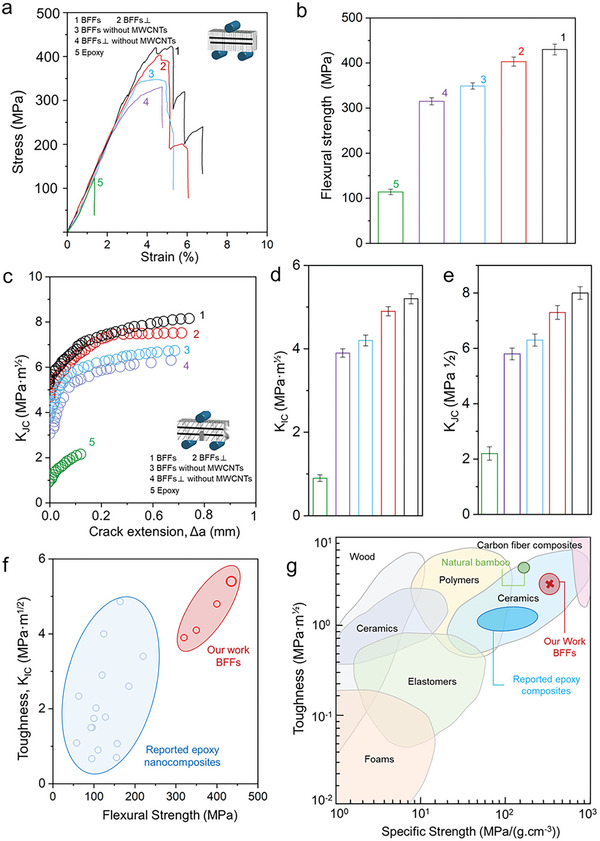
Mechanical properties of the BFFs and in comparison with relevant materials. a) Experimental flexural stress–strain curves; b) flexural strengths; c) fracture resistance (R‐curve) behaviors from notched flexural tests; d) fracture toughness for crack initiation (*K*_IC_); e) stable crack propagation (*K*_JC_); f) comparison of the strength and toughness properties of the BFFs with those of existing resin‐based composite materials; g) comparison of the specific strength and toughness (*K*_IC_) of the BFFs with those of other engineering materials in the Ashby map.

The results of the three‐point bending test, where the force is applied perpendicular to the CF axis, are presented in **Figure** **3**a,b. It is noteworthy that the bending strength of the BFFs reaches 430.8 MPa, surpassing that of the BFFs⊥, which is 398.5 MPa. The bending strengths of the BFFs and the BFFs⊥ are higher than those of the BFFs and the BFFs⊥ without MWCNTs. On the other hand, the fracture toughness at the onset of crack propagation (*K*_IC_) for BFFs is ≈5.4 MPa m^1/2^, while the fracture toughness at stable crack propagation (*K*_JC_) is nearly 8.3 MPa m^1/2^ (Figure 3c–e). The fracture toughness values exceed those of the BFFs and the BFFs⊥ without MWCNTs. Thus, the BFFs are outstanding in attaining both strength and toughness, and the BFFs and the BFFs⊥ show superior mechanical properties compared to those without MWCNTs. These indicate that cellular network featuring cell faces bridging the CFs, along with the incorporation of MWCNTs, contributes to the enhanced bending strength and fracture toughness of the BFFs. Additionally, Figure S8 (Supporting Information) presents the results of the three‐point bending strength and toughness tested in the direction separating the CFs. Notably, the bending strength of the BFFs measures 299.8 MPa, which exceeds the bending strength (250.2 MPa) of the BFFs⊥. The incorporation of MWCNTs in the BFFs system contributes to higher bending strength and toughness compared to those without MWCNTs.

When comparing the mechanical properties of the BFFs to those of existing epoxy resin‐based composite materials (Figure 3f; Table S2, Supporting Information), the BFFs show remarkably higher strength and toughness. The material density of BFFs is 1.27 g cm^−3^, which is similar to that of epoxy resin matrix composites, and the BFFs exhibit higher strength than natural bamboo (Figure 3g and Table S2, Supporting Information). Moreover, the specific strength and toughness performance of the BFFs are comparable to most ceramic‐based composite materials (Figure 3g; Table S2, Supporting Information). In comparison to continuous CF composites with a carbon fiber volume fraction of ≈56%, the BFFs exhibits a flexural strength of 430.8 MPa at a lower carbon fiber volume fraction of 1.5–2%. Although it does not reach the strength levels of high‐volume continuous carbon fiber composites, BFFs showcases good fracture toughness (*K*_IC_) compared to certain counterparts (Figure 3g; Table S2, Supporting Information). The mechanical properties of BFFs are even better than synthetic bioinspired composite materials, for example, nacre‐like,^[^ ^25^, ^26^, ^27^ ^]^ bone like,^[^ ^28^ ^]^ and bouligand like composites.^[^ ^29^ ^]^ Furthermore, the mechanical properties achieved in this study are compared with available data from similar carbon‐reinforced epoxy‐matrix composite materials reported in the literature (Table S3, Supporting Information). Existing studies can be considered utilizing two main strategies: one incorporating CNTs and graphene as the reinforcing materials, and the other focusing on special internal structural design of the reinforcement. The comparison reveals that composite materials with a face‐bridging fiber reinforcement structure, like the BFFs, exhibit superior toughness and strength compared to most nano‐particle reinforced epoxy‐based composites. Furthermore, the simultaneously high strength and toughness achieved by the BFFs surpass that of resin‐based composites reinforced with materials other than graphite type material, including wood scaffolds,^[^ ^30^ ^]^ clay,^[^ ^31^ ^]^ MXene.^[^ ^27^, ^32^ ^]^ Therefore, the developed bamboo‐inspired crack‐face bridging fiber reinforced composites, through successfully replicating the symbolic structure of bamboo, demonstrate excellent mechanical properties that exceed those of engineering resin‐based nanocomposites and bioinspired structural composites.

### 2.3 Strengthening and Toughening Mechanisms of the BFFs

**Figure 4:**
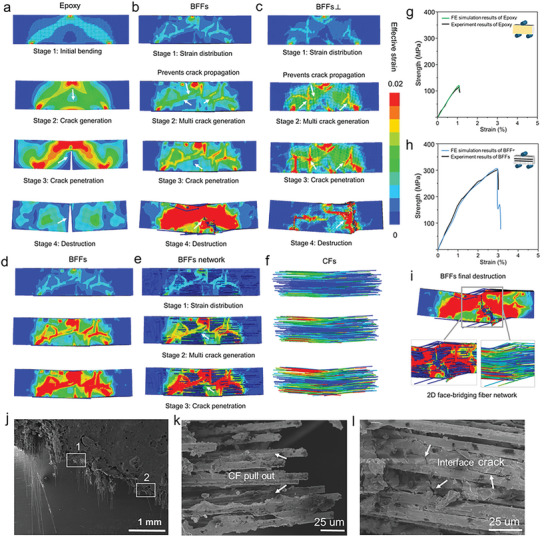
Damage and failure mechanism analyses in three‐point bending test. The fracture behavior obtained from finite element (FE) simulation: a) epoxy resin; b) BFFs; c) BFFs⊥. Strain distributions of different components in the BFFs: d) strain distribution in the overall BFF; e) strain distribution in the cellular network that bridges CFs; f) perspective view of the CFs. Comparison of bending stress–strain curves from FE simulation and those from experiments: g) epoxy resin; h) BFFs. i) Analysis of final failure surface in FE simulation; j) SEM image of BFFs failure surface; k) SEM image zoomed in at point 1 in (j); l) SEM image zoomed in at point 2 in (j).

Comparing the results of finite element (FE) simulations for the bending behavior of pure resin and the bamboo‐inspired crack‐face bridging fiber reinforced composites reveals interesting strengthening and toughening mechanisms of the BFFs (**Figure** **4**; Figure S10, Supporting Information). First, the simulated stress–strain and failure behaviors of the BFFs and the BFFs⊥ and the pure resin agree well with those experimental results, confirming the reliability of the FE simulations, as depicted in Figure 4g,h. Analyzing the deformation process, the failure mode of the pure resin is primarily brittle fracture, as shown in Figure 4a. In contrast, the BFF composite exhibits smaller strains at each stage and a stepwise failure pattern during bending, indicating its strong ability to sustain load and deflect crack, as depicted in Figure 4b,c. Figure 4d,e illustrates that initially, the main load is borne by the cellular network with faces bridging the CFs, and the load is effectively dispersed along this network. In addition to this superior loading‐sustaining ability, the cell faces effectively transfer forces to the stiffer CFs, which contributes to retarding the initiation and propagation of cracks (Figure 4f). Likewise, the three‐point bending FE simulation in the direction separating the CFs (Figure S11, Supporting Information) reveals that upon loading, stress is efficiently transmitted and dispersed along the cellular network within the BFFs. These structure‐induced strengthening mechanisms lead to the significantly high bending strength of the BFFs.

The simultaneously superior high toughness of the BFFs arises from the favorable damage and failure behaviors resulting in a stepwise failure pattern (Figure 4i–k) and the larger deformability before final failure (Figure 4b). The CF‐bridging cellular network that interfaces CFs and epoxy allows a plenty of sites for crack initiation (microcracking, Figure 4b,d,e,i); this also results in a zigzag cracking path (Figure 4i) giving rise to crack twisting and deflection (Figure 4j,l) and CF pullout and fracture failure (Figure 4j,k). The final fracture mode of BFFs⊥ displayed a stepped fracture pattern (Figure S12a, Supporting Information), accompanied by CF pullout and interface fracture (Figure S12b,c, Supporting Information), underscoring the cellular carbon network's role in controlling the toughening crack path. These damage and failure mechanisms dissipate a large amount of energy, which contributing to the high toughness. In the other loading direction in three‐point bending (Figure S11, Supporting Information), the final fracture mode also exhibits a stepped fracture pattern, indicating the role of the cellular network in controlling the cracking path for toughening. Thus, the deformation and failure behaviors of the BFFs through characterization and FE analyses highlight the distinct, advantageous load‐sustaining and load‐transferring capabilities as well as cracking mechanisms due to the bamboo‐inspired CF‐bridging cellular network microstructure.

**Figure 5:**
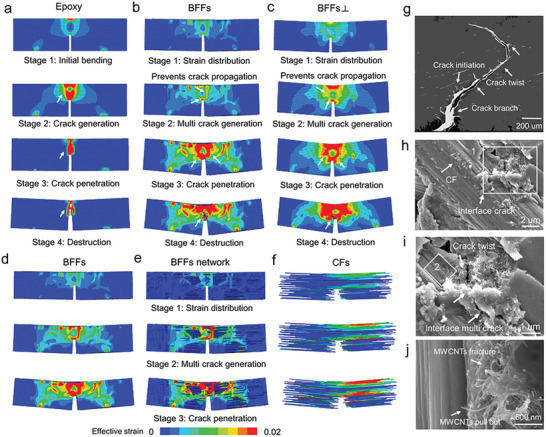
Fracture toughness behaviors through notched three‐point bending analyses. FE simulation results of fracture testing process: a) epoxy resin; b) BFFs; c) BFFs⊥. The strain distributions of different components in the BFFs in FE simulation: d) strain distribution in the overall BFF; e) strain distribution in the cellular network that bridges CFs; f) perspective view of the CFs. g) Failure behavior of notched three‐point bending test specimen of the BFFs; h) SEM image of the interface fractures between the cellular network faces and the resin; i) zoomed‐in SEM image at point 1 in (h); j) zoomed‐in SEM image at point 2 in (i).

Comparing the fracture toughness behaviors of notched pure epoxy resin and the BFFs reveal the crack‐face bridging structure induced toughening mechanisms. For pure epoxy resin (**Figure** **5**a), a crack propagates linearly from the notch tip and leads to catastrophic failure, a characteristic behavior of brittle materials. In contrast, the BFFs exhibit a different failure pattern. As shown in Figure 5b,c, the crack paths are more tortuous and spread over large volumes, making it more difficult for cracks to propagate and to cause sudden fracture. Despite the crack formation at the notch tip initially, strains are delocalized and distribute over larger volumes throughout subsequent loading, as many branches and small cracks evolve around the main crack (Figure 5b–f). The crack propagates along a tortuous path, deflected by the 2D faces of the cellular network (Figure 5e,f). The bridging faces connecting to CFs also effectively transfer load to the CFs, which contributes to superior load‐bearing ability and delaying failure. Finally, the crack continues to extend until the specimen fractures completely (Figure 5d–f).

The zigzag crack deflection observed in the BFFs is a critical external toughening mechanism, akin to those found in bioinspired structural materials with high fracture resistance.^[^ ^33^, ^34^, ^35^ ^]^ The SEM observations reveal such toughening mechanisms occurring at multiple length scales, including crack deflection, crack branching, and crack bridging (Figure 5g). The crack deflection process under higher magnifications (Figure 5h,i) shows that the interfaces between the cellular network faces and the resin contributes to crack twisting and formation of multiple microcracks. This mechanism effectively alleviates local excessive stress concentration and consumes a significant amount of energy. Similarly, in the notched three‐point bending simulation loaded to separate the CFs (Figure S11, Supporting Information), a step‐like fracture pattern is observed, with cracks being deflected at the cellular network and forming additional branches. These results demonstrate that the incorporation of the cellular network, which bridges the CFs through cell faces significantly enhances toughness through strain delocalization, crack branching, and crack deflection, as well as higher resistance to crack propagation and failure. BFF exhibits a higher storage modulus compared to epoxy resin (Figure S12, Supporting Information). The increase in storage modulus can be attributed to the rigid interface, which effectively restricts the mobility of epoxy resin molecules within the interface region, thus enhancing the interfacial adhesion and stress transfer between the porous cellular carbon network reinforcement and the resin interface. This enhanced toughness is crucial in preventing catastrophic failure and increasing the overall durability of the composite material.

In addition, the incorporation of MWCNTs into the faces of the cellular network plays an important role in synergistic strengthening and toughening. Nanoscale MWCNTs serve as the reinforcing phase in the faces of the cellular carbon network, thus enhancing the load‐bearing ability of the cell faces (Figure S15, Supporting Information). Besides, the extraction and fracture of MWCNTs within the carbon network can be observed, accompanied by a corresponding step‐like fracture pattern at the interface (Figure 5j). This indicates that MWCNTs contribute to energy dissipation and enhance the fracture toughness of the overall composite material, which explains the highest bending strength and fracture toughness exhibited by the BFFs containing MWCNTs.

**Figure 6:**
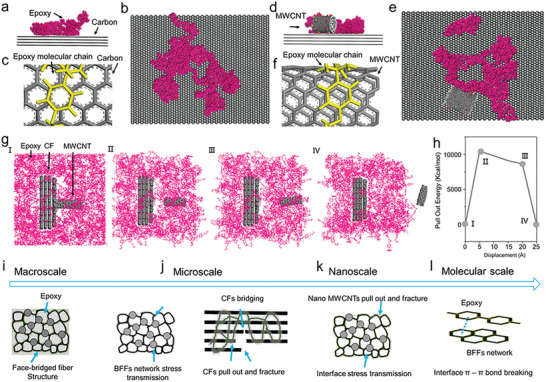
Molecular dynamics (MD) simulation analysis of the interface reactions and the overall strengthening and toughening mechanisms: a) adhesion behavior at the epoxy‐cellular network interface, b) top view of (a), c) π–π bond forming between the epoxy and the network, d) adhesion behavior at the epoxy‐cellular network interface with a MWCNT in the system, e) top view of (d), f) π–π bond forming between the epoxy and the MWCNT outer wall in the network, g) process of pulling MWCNT out from the interface, h) pull out energy variation during the pull out process. Multiscale strengthening and toughening mechanisms: i) from the macroscale, the intrinsic characteristics of the CFs, the cellular MWCNTs‐containing carbon network, and the epoxy that compose the BFFs contribute to the overall superior loading‐bearing and deformation capabilities, j) at the microscale, the cellular network that bridges CFs through cell faces not only transfers load to the CFs but also sustains load, contributing to the strengthening, while the strain delocalization, crack branching, and crack propagation effectively restrict crack propagation and dissipate energy to enhance toughness, k) at the nanoscale, MWCNTs pullout and fracture at the interface increases the strength and delays crack initiation and propagation to increases the toughness, l) at the molecular scale, breaking the π–π bonds between the epoxy and the cellular network/MWCNTs that consumes sufficient amount of energy is one key of the strengthening and toughening effects.

The adsorption model of MD simulation is employed to further elucidate the strengthening and toughening mechanisms. **Figure** **6**a,b shows the adhesion behavior at the epoxy‐cellular network interface. The distance between the six‐membered ring in the epoxy molecule and the centroid of the carbon is found to be 4.3–4.5 Å, indicating π–π bond interactions.^[^ ^36^ ^]^ Epoxy demonstrates stable adsorption on the carbon surface, with the six‐membered rings in the epoxy forming a stable conformation with the six‐membered rings of carbon primarily through π–π interactions (Figure 6c). From Figure 6d,e, it can be seen that in the presence of MWCNT, the epoxy resin can stably adsorb onto the outer walls of the MWCNT. The interaction of π–π bonds facilitates the formation of a stable structure between the six‐membered rings of epoxy and the MWCNT's six‐membered ring (Figure 6f). It is noteworthy that observable shifts have occurred in the D and G frequency bands following the modification (Figure S16, Supporting Information). The D band has shifted by 7 cm^−1^ units to the left, while the G band has shifted by 23 cm^−1^ units to the left. These shifts further confirm the existence of interface π–π interactions between the cellular carbon network and the resin.^[^ ^37^ ^]^

MD simulations of MWCNT pull‐out from the interface show an increase in pulling energy as the MWCNT is extracted (Figure 6g,h). When the MWCNT is completely pulled out, the pulling energy tends to zero. This increase in energy during the pull‐out process can be attributed to the disruption of the π–π interactions between the carbon network and the resin. The pull‐out of MWCNT from the cell faces consumes energy. The SEM images also provide evidence of pulling MWCNTs out (Figure 5h–j), which dissipates energy and alters crack path, validating the effectiveness of the simulations. These MD simulation results reveal the importance of π–π interactions in the stable adsorption of epoxy resin on carbon surface and MWCNTs, and demonstrate the role of energy consumption during the pullout of MWCNT from the cell faces in dissipating energy and influencing crack deflection.

Based on the above analyses, the BFFs show outstanding mechanical performance due to mechanisms operating at multiple length scales. At the macroscopic scale (Figure 6i), compositing the CFs that are face‐bridged via a cellular carbon network and the epoxy resin leverages respective advantageous mechanical properties and meanwhile introduces new structural strengthening and toughening mechanisms. At the microscale (Figure 6j), the 2D faces of the cellular network, which bridge the CFs, effectively transfer load to the CFs and also sustain load throughout the composite system, delaying occurrence of failure. The strain delocalization, crack branching, and crack propagation arising from the bamboo‐inspired CF‐bridging cellular network microstructure are core to the high toughness and strength of the BFF composites. The extraction, fracture, and bridging of the CFs at the interface effectively prevent crack initiation, contributing to the enhanced strength and toughness. At the nanoscale (Figure 6k), the pulling out and fracture of MWCNTs in the faces of the cellular network play a crucial role in crack deflection and load absorption. These processes enhance the material's ability to withstand external forces and dissipate energy. Furthermore, the rupture of the interface formed by π–π interactions between the epoxy resin and the six‐membered rings in the carbon network also contributes to the overall strength and toughness of the composite (Figure 6l).

## 3 Conclusion

In this work, a bamboo‐inspired crack‐face bridging fiber reinforced composite is developed by creating a cellular carbon network connecting the CFs within the epoxy resin. The fabricated composites are both strong and tough, exhibiting a high strength of 430.8 MPa and an impressive fracture toughness of 8.3 MPa m^1/2^ with the cell faces aligned parallel to the CFs and containing MWCNTs, which exceed most reported values of resin‐based nanocomposites. The BFF composites show interesting structural strengthening and toughening mechanisms originated from the bamboo‐inspired structure featuring cell faces bridging CFs and containing MWCNTs, which differ from the 1D fiber bridging in conventional fiber reinforced composites. The 2D faces of the cellular network effectively transfer load to the CFs and also sustain load, contributing to strengthening and delaying failure. The strain delocalization, crack branching, and crack deflection lead to a step‐like failure pattern, consuming a large amount of energy and resulting in an exceptionally high fracture toughness. At the nanoscale, the pulling out and fracture of MWCNTs promote crack deflection and load absorption, and the pull‐out of the carbon network from the resin disrupting the π–π bonding also enhances the overall strength and toughness. The results and finding provide a new strategy that is effective and efficient to create strong and tough bioinspired composite materials, overcoming the either‐strength‐or‐toughness trade‐off in conventional carbon reinforced resin‐based composites for upgrading and stimulating further development of novel bioinspired high‐performance composites.

## 4 Experimental Section

Any additional information relevant to the materials methodology is included in the Supporting Information.

## Conflict of Interest

The authors declare no conflict of interest.

## Author Contributions

Conceptualization: H.W.; Methodology: H.W., Z.Y.W., J.T., B.W., and C.B.H.; Software: H.W. and Z.Y.W.; Investigation: H.W., Z.Y.W., J.T., and C.B.H.; Supervision: J.T. and C.B.H; Writing—original draft: HW; Writing—review & editing: J.T., Z.Y.W., B.W., and C.B.H.

## Supporting information

Supporting Information

## Data Availability

Data sharing is not applicable to this article as no new data were created or analyzed in this study.
