# Supplementary material for: Bamboo‐Inspired Crack‐Face Bridging Fiber Reinforced Composites Simultaneously Attain High Strength and Toughness
Source: Adv Sci (Weinh). 2023 Dec 28;11(10):2308070. doi: 10.1002/advs.202308070 (PMC10933601; doi:10.1002/advs.202308070)
Supplement: Supplementary file 1 — Supporting Information [file ADVS-11-2308070-s001.pdf]

ADVANCED SCIENCE
Open Access

Supporting Information

for *Adv. Sci.*, DOI 10.1002/advs.202308070

Bamboo-Inspired Crack-Face Bridging Fiber Reinforced Composites Simultaneously Attain High Strength and Toughness

Hao Wang, Zhangyu Wu, Jie Tao*, Bin Wang* and Chaobin He**

Supporting information

Bamboo-inspired crack-face bridging fiber reinforced composites simultaneously attain high strength and toughness

Hao Wang[1,2], Zhangyu Wu[3*], Jie Tao[4*], Bin Wang[2*], Chaobin He[1, 5*]

[1] Department of Materials Science and Engineering, National University of Singapore, Singapore

[2] Department of Mechanical Engineering, City University of Hong Kong, Hong Kong

[3] School of Materials Science and Engineering, Southeast University, China

[4] School of Materials Science and Technology, Nanjing University of Aeronautics and Astronautics, China

[5] Institute of Materials Research and Engineering, Agency for Science Technology and Research (A*STAR), Fusionopolis Way, Innovis, Singapore

*Corresponding Authors: wuzy@seu.edu.cn, taojie@nuaa.edu.cn, binwang55@cityu.edu.hk, msehc@nus.edu.sg

Material and Methods

Experimental

Materials

Carbon fiber (CF) tow was purchased from Weihai Guangwei Composite Material Co., Ltd. China (TZ700S-12K, Unsized). The MWCNTs (with a purity of 95%, a length of 0.5-2 um, and a diameter of 8-15nm) were received from Nanjing XFNANO Materials Tech (Nanjing, China). Chitosan was selected as the carbon precursor of the cell network structure. It is a medium molecular weight (C804726, deacetylation degree >75%, viscosity: 200 - 800 cps, Macklin Biochemical Company) converted from chitin in crab shells through deacetylation reaction. The epoxy resin (DGEBA) and curing agent (DDS) were procured from Zaoqiang County Shengpeng Trading Co., Ltd., China.

Fabrication process of Bamboo-inspired crack-face bridging fiber reinforced composites (BFFs)

The CS solution was prepared by the addition of 2 g of CS powder to 100 mL of deionized water with 2% (v/v) acetic acid, followed by even stirring and standing until no bubbles were observed, and the solution was transparent to afford a 2 wt% CS solution. The mass ratio of the reinforcement (CFs and MWCNTs in a weight ratio of 4.5:0.5) to the CS solution was 5:95. Initially, 1 g of CS powder was combined with 0.5 wt% of MWCNT and added to 50 mL of water. Ultrasonic stirring was employed for a duration of 2 hours until a uniform dispersion was achieved, free from any observable agglomeration. Subsequently, a 2 vol% acetic acid solution was added to produce the final CS-MWCNT solution. Second, the 2D bridging of 1D reinforced honeycomb-like network was constructed. A CF tow-spreading process [1] was used to turn 12K CF filaments into a thin ply to prepare a low-volume-fraction CF (Figure 1), and CFs with a volume fraction of 1.5-2% were wound-passed though the CS-MWCNT solution at a uniform speed, which were then evenly wound on a cylinder with a length of 10 mm and a diameter of 2 mm. When the sample on the cylinder was completely wound, the sample was cut from the cylinder, followed by rolling. Then, it was put into a PU plastic box (length 5 cm × width 5 cm × height 8 cm) and freeze-casted and dried at −60 °C for 24 h. The freeze-casting direction was along the length of the CF. The obtained CF

scaffolds were subjected to heat-treatment at temperatures ranging from room temperature to 550°C and maintained for 2 h. Next, it was infiltrated by the epoxy resin followed by curing, and the obtained sample was denoted as “BFFs.” For comparison, the BFFs without MWCNTs also were fabricated, and the freeze-casting direction was adjusted to be perpendicular to the length of CF. The sample obtained by the freeze-casting direction perpendicular to the CF was denoted as “BFFs⊥” The sample without MWCNTs and the freeze-casting direction perpendicular to the CF was denoted as “BFFs⊥ without MWCNTs” The sample without MWCNTs and the freeze-casting direction along the CF was denoted as “BFFs without MWCNTs.”

Sample characterization

The thermal stability of the bamboo-inspired crack-face bridging fiber reinfored composite materials (BFFs) was investigated by thermogravimetric analysis (TGA) (100-240V/50-60Hz, PerkinElmer). The tests were carried out in a N_2 atmosphere of 50 ml/min and a heating rate of 10 K/min in the temperature range from 25 °C to 800 °C. The surface of the samples was analyzed by acquiring Raman spectrometer from LabRAM (Perkin Elmer). The samples were meticulously sectioned into pieces with dimensions of approximately d = 1–1.5 mm in thickness, b = 2 mm in width, and l = 20–25 mm in length. Subsequently, they underwent a polishing process using sandpaper to eliminate edge defects. In the three-point bending test (at least three samples), a support span of 10 mm was employed, the loading point size (hemispheric radius) was approximately 3.2 mm, and the bending displacement rate was set at 0.5 mm/min. For single-edge notch bending (SENB) testing, specimens with dimensions of d ≈ 1 mm and b ≈ 2 mm were notched to approximately 50% of their width using a 150 µm thick diamond blade. The notches were subsequently meticulously refined by repeatedly swiping with a knife blade, resulting in a final notch radius of approximately 30 µm. SENB testing, which involved a minimum of three samples, was conducted at a consistent displacement rate of 0.05 mm/min. Both the three-point flexural strength and SENB tests were performed using a universal testing machine (Instron 5689, Instron Corp., USA). The three-point bending test followed the guidelines provided by ASTM D790-03. In compression tests, each sample was compressed at a rate of 1 mm/min using a Instron 5689, Instron Corp., USA

tension/compression machine. A minimum of three samples were tested.

Surface morphology was characterized by using a field-emission scanning electron micro-analyser (TESCAN LYRA3, Bruker Nano Berlin, Germany). The DMA test was conducted using a dynamic mechanical thermal analyzer (TA Q800, USA) in a three-point bending mode. The samples were subjected to testing at a frequency of 1.0 Hz, over a temperature range of 40-290°C, with a heating rate of 10°C/min. The distribution of MWCNTs in the BFFs were imaged using a high-resolution transmission electron microscope (HRTEM, JEM-2100F, JEOL Japan).

The stress (σ), and strain (ε) using the three-point bending force–displacement curves are calculated by the following equations:

$$\sigma = \frac{3\mathrm{FS}}{2bd^2} \tag{S1}$$

$$\varepsilon = \frac{6\mathrm{Dd}}{\mathrm{S}^2} \tag{S2}$$

where F is the force at the point of failure, D is the displacement at the point of failure, b and d represent the specimen's width and thickness, S is the length of the support span.

The fracture toughness, K_{IC}, under plane strain conditions is calculated using the following equations[2]：

$$K_{IC} = \frac{P_{IC} f(a/W)}{BW^{3/2}},\ x = a/W \tag{S3}$$

$$f(x) = \frac{3a/W^{1/2}\left[1.99 - x(1-a/W)\left(2.15 - 3.93a/W + 2.7(\frac{a}{W})^2\right)\right]}{2(1+2a/W)(1-a/W)^{3/2}} \tag{S4}$$

where P_{IC} is the maximum load, B and W are the width and height of the specimen, and a is the initial crack length.

The fracture toughness, K_{JC}, was assessed using J-integral calculation, which accounts for both the elastic and plastic contributions. This approach is in line with previously established methods utilized for estimating the properties of various inspired composites.

$$\mathrm{J} = J_{el} + J_{pl} \tag{S5}$$

where J_{el} is the elastic contribution on the basis of linear elastic fracture mechanics,

$$J_{el} = \frac{\mathrm{K}_{IC}^2}{E'} \tag{S6}$$

where the plastic contribution, J_{pl} can be calculated with the following equation:

$$J_{pl} = \frac{2A_{pl}}{B(W-a)} \tag{S7}$$

where A_{pl} is the plastic area underneath the load–displacement curve, J values can be transformed into K values by the following equation:

$$K_{JC} = (JE')^{1/2} \tag{S8}$$

in which $E' = E(1 - v^2)$, where E represents Young's modulus and v denotes the Poisson ratio. It should be noted that the influence of the variation in E on K_{JC} is relatively limited. Therefore, in this context, E' can be effectively replaced by E.

The SENB method leverages the equivalence between compliance and crack length [2] to analyze toughness. This is accomplished by computing compliance using the formula $C = \frac{u}{f'}$, where u represents crack propagation, and f' denotes the forces at each point after the crack has propagated beyond that point. Subsequently, a recursive process is applied to calculate the crack length, as follows:

$$a_n = a_{n-1} + \frac{W-a_{n-1}}{2} \times \frac{c_n - c_{n-1}}{c_n} \tag{S9}$$

$$c_n = \frac{u_n}{f_n} \tag{S10}$$

$$\Delta a = a_n - a \tag{S11}$$

where W is the width of the sample, a and c are the crack length and compliance, respectively calculated at the n and $n-1$ steps, and Δa is the amount by which the crack extends.

Molecular dynamics analysis

Molecular dynamics (MD) simulation was conducted to construct an interface simulation model and reveal the strengthening and toughness mechanisms of BFFs. For the bamboo-bioinspired carbon network surface model with MWCNT, the four-layer graphene model was used (**Figure S1a**) [3, 4] (the size was 120 Å × 90 Å, and the layer spacing was set at 3.35 Å). Based on our previous research work [4] to model the epoxy resin, a mixture of diglycidyl ether of bisphenol A (DGEBA) and 4,4'-diaminodiphenylsulfone (DDS) curing agent was prepared at a ratio of 2:1. The initial cutoff distance was set at 0.35 nm and the maximum cutoff distance

was 0.7 nm. The mixture was then cross-linked to achieve a degree of crosslinking of 80%. Subsequently, molecular dynamics (MD) simulations were performed to minimize the system energy and relax the model (**Figure S1a**). The three-walled armchair CNT was adopted to represent MWCNT in the simulation (**see Figure S1c**) [5] (the CNT wall separation was set to 0.35 nm). We placed the MWCNT on the surface of graphite to create a representation of a 2D bridging of 1D reinforced honeycomb-like carbon network. The epoxy were filled on the surfaces of carbon in a box (the size was 120 Å × 90 Å, and the density was 0.4 g/cm^3). Then, the interface simulation models were obtained, referred to as BFFs (**Figure S1a**) and BFFs without MWCNTs (**Figure S1b**).

A condensed-phase optimized molecular potentials for atomistic simulation studies [6] (COMPASS) force field was adopted to model structural optimization and to calculate interface mechanical properties. The CF interface model was optimized in the canonical ensemble (NVT) dynamics at 298 K (**Figure S2**).

The Nosé-Hoover thermostat and Andersen barostat [7] were selected to control the temperature and pressure, respectively. The van der Waals interaction energy was calculated by the Lennard-Jones potential [8, 9] with a cut-off distance of 12.5 Å. The Newtonian equations of motion were solved using the Verlet velocity-time integration algorithm with a time step of 1.0 fs [9]. The interface models were optimized, and dynamics were relaxed under a canonical ensemble molecular dynamics (NVT-MD) simulation with a time step of 1.0 fs.

Interfacial pull out energy was used to measure MWCNT pull out process at the interface, which can be calculated as follows:

$$\Delta \mathrm{E} = E_{total} - \left(E_{carbon} + E_{Epoxy}\right) \tag{S12}$$

where $\Delta \mathrm{E}$ is the pull out energy at the interface, E_{total} is the total potential energy of the system, E_{carbon} is the potential energy of the carbon scaffold and E_{Epoxy} is the potential energy of the epoxy.

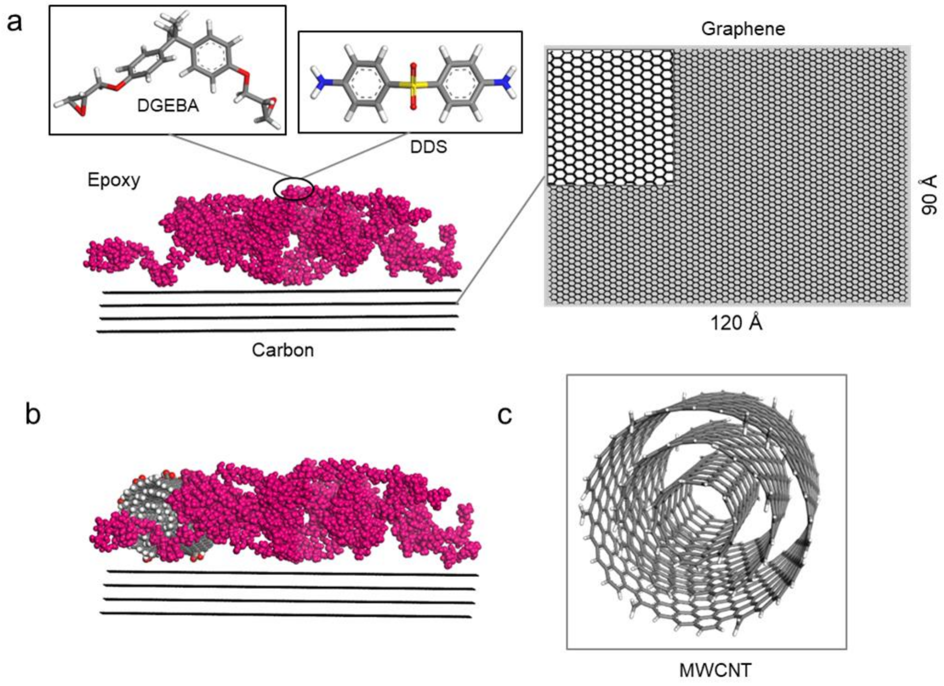

Figure S1 MD simulation modeling: **a** BFFs without MWCNT; **b** BFFs; **c** MWCNT.

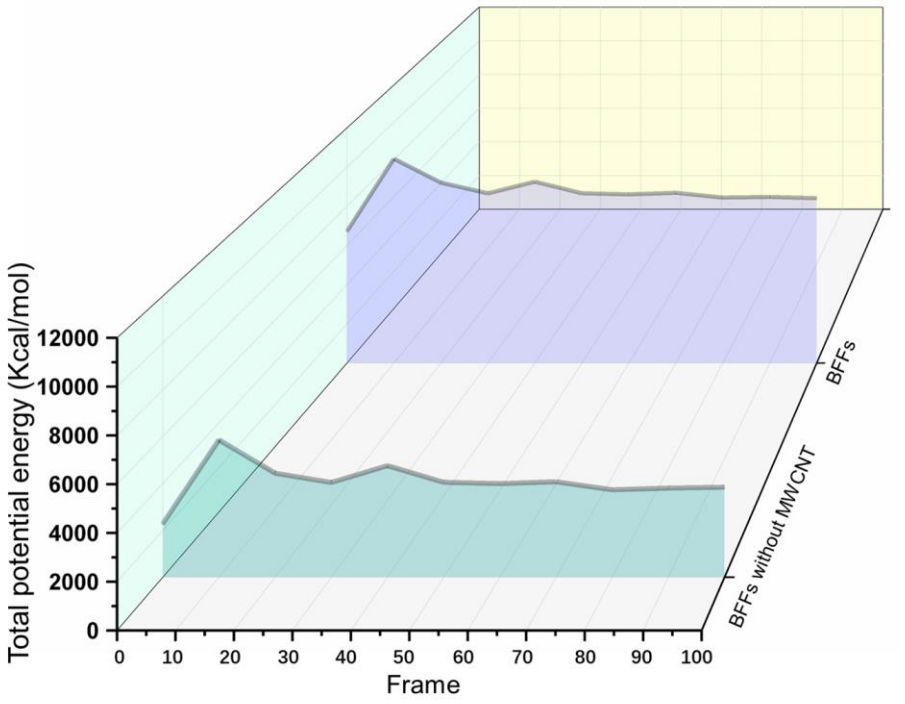

Figure S2 Relationship between the total potential energy and frame during MD optimization.

Finite Element Simulation

Straight fiber models

Using a self-programmed script, the 3D straight fibers with certain diameters randomly

dispersed in a spatial domain can be generated in the ANSYS software. The spatial location of each straight fibers was determined by the two endpoints (Equation S13), and the spatial orientation of fiber model can be defined by Equation S14. By adjusting the angle parameters of fibers, the oriented fibers align with different axis can be generated. **Figure S3** presents the 3D straight fiber models that is oriented along with the Z axis, with the fiber diameter and the content set to be 7μm and 2% by volume, respectively.

$$\begin{pmatrix} X_2 \\ Y_2 \\ Z_2 \end{pmatrix} = \begin{pmatrix} X_1 \\ Y_1 \\ Z_1 \end{pmatrix} + \begin{pmatrix} X_p - X_m \\ Y_p - Y_m \\ Z_p - Z_m \end{pmatrix} \tag{S13}$$

where (X_1, Y_1, Z_1) is the initial coordinate of a fiber, (X_m, Y_m, Z_m) is the initial coordinate of mid-point of a fiber, (X_p, Y_p, Z_p) is the coordinate of a random point in the specimen, and (X_2, Y_2, Z_2) is the new coordinate of a fiber.

$$Ori\_2 = Ori\_1 \begin{pmatrix} \cos\beta\cos\gamma & \cos\beta\cos\gamma & -\sin\beta \\ \sin\alpha\sin\beta\cos\gamma - \cos\alpha\sin\gamma & \sin\alpha\sin\beta\sin\gamma + \cos\alpha\cos\gamma & \sin\alpha\cos\beta \\ \cos\alpha\sin\beta\cos\gamma + \sin\alpha\sin\gamma & \cos\alpha\sin\beta\sin\gamma - \sin\alpha\cos\gamma & \cos\alpha\cos\beta \end{pmatrix} \tag{S14}$$

where ***Ori_2*** and ***Ori_1*** respectively denotes the final and initial orientation of fiber, $\boldsymbol{\alpha}$, $\boldsymbol{\beta}$ and $\boldsymbol{\gamma}$ respectively represents the angle with respect to coordinate axis, that is, ***X***, ***Y*** and ***Z*** axis.

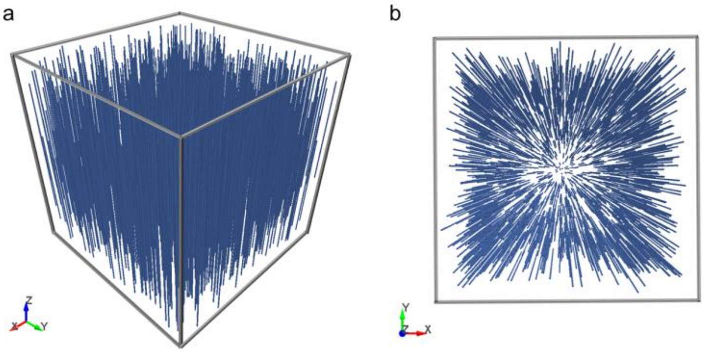

Figure S3 3D straight fiber models with a diameter of D_f randomly dispersed within a spatial specimen domain, which is oriented along with Z axis. **a** An isometric view, **b** a top view.

3D mesoscale model

To effectively replicate the spatially ordered pore structure in the BFFs, a novel 3D particle model with random shape configurations to simulate those pores was developed. The generation process of a 3D particle model is exhibited as **Figure S4**. From **Figure S4a**, a random quadrilateral *ABCD* inscribed a circle with a diameter of D_a is first generated, and the internal angle could be calculated according to the side length as Equation S125. Subsequently,

a random octahedron *EF-ABCD* is created, as depicted in **Figure S4b**, utilizing the above-generated quadrilateral *ABCD*. After that, according to the vector-driven growth method (see **Figure S4c**), a random decahedron *EFG-ABCD* (see **Figure S4d**) could be generated based on the generated octahedron *EF-ABCD*. The detail steps of the vector-driven growth method are introduced as follows:

Step 1: Selecting the longest edge in the octahedron *EF-ABCD* to determine a random point to be the seed for the following growth procedure.

Step 2: Generating an outward vector Vij = Vi + Vj built upon the normal vectors (Vi and Vj) of the adjacent planes of the longest edge. Along the vector Vij, a new vertex *G* is then created by randomly adjusting the growth parameter that is related to the spatial coordinates of the newly generated vertex.

Step 3: By connecting all the vertexes, a random decahedron *EFG-ABCD* is generated.

It should be noted that there is a correlation relationship between the surface number and the random growth time, that is, a random polyhedron with a surface number of $2N+8$ could be generated after N times of random growth derived from a random octahedron. Thus, 3D random particle models with 256 surfaces can be generated after 124 times of random growth, as shown in **Figure S4e**. Afterwards, those generated particle models are randomly delivered and compacted in a spatial domain, and the spatially ordered pore structures were established as shown in **Figure S4f**, which highly agree with the SEM results of the bioinspired composite, indicating the feasibility of the generated 3D mesoscale model in this work.

$$\begin{cases} \operatorname{Cos} A = \dfrac{d^2 + c^2 - a^2 - b^2}{2(d \cdot c + a \cdot b)} & \text{(a)} \\ \operatorname{Cos} B = \dfrac{d^2 + a^2 - b^2 - c^2}{2(a \cdot d + b \cdot c)} & \text{(b)} \\ \operatorname{Cos} C = \dfrac{a^2 + b^2 - c^2 - d^2}{2(a \cdot b + c \cdot d)} & \text{(c)} \\ \operatorname{Cos} D = \dfrac{c^2 + d^2 - b^2 - a^2}{2(c \cdot d + a \cdot b)} & \text{(d)} \end{cases} \qquad \text{(S15)}$$

According to the 3D mapping meshing method, the random pore structure was meshed using the hexahedron elements. While the thin layer randomly wrapped on the pore phase was also generated for modeling the network phase. The average thickness of the network structure was set to be 1/8-1/5 of the attached pore size. According to the present experiment, those pores

were filled by the resin matrix, thus, the material attribute of the pore phase were determined by the physical and mechanical properties of resin matrix. And the interfacial relationship between resin matrix and network were modeled using the bonding assumption. **Figure S4b-c** depicts a 3D two-phase finite element model of cube specimen, which is composed of the random pore phase (marked in green) and the network phase (marked in gray). The pore size ranges from 0.5mm to 2mm, and the mesh size is set to 0.1mm.

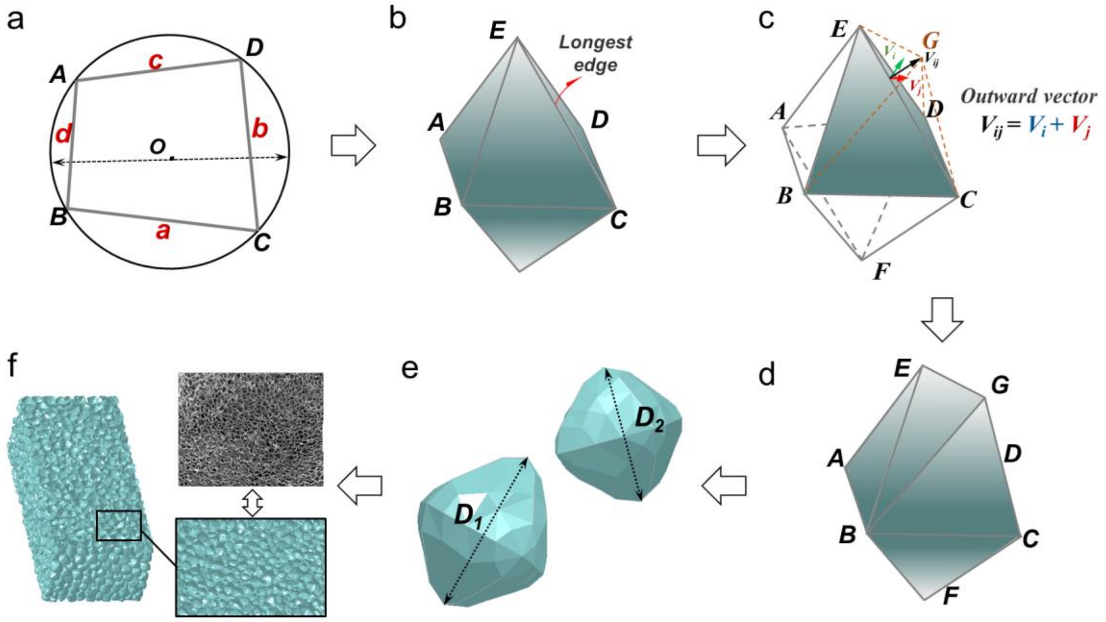

Figure S4 Generation process of 3D particle models with random shape and size configurations. **a** a random quadrilateral *ABCD* inscribed a circle, **b** a random octahedron *EF-ABCD* generated from the quadrilateral *ABCD*, **c** and **d** a random decahedron *EF-ABCD* is generated based on the octahedron *EF-ABCD*, **e** 3D random particle models with 256 surfaces; **f** a 3D random particle assembly for simulating the spatially ordered pore structures in the BRCs.

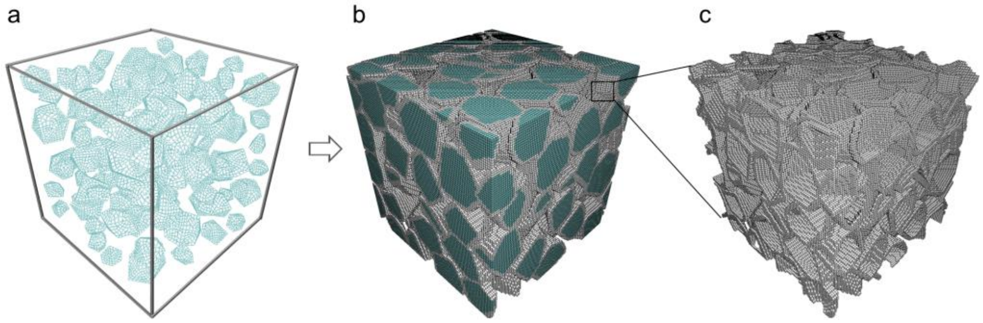

Figure S5 Establishment of 3D mesoscale model of cube specimen containing 3D random orientated pores represented by 3D random particle models. The corresponding 3D two-phase finite element model in (**a**) is composed of the random pore phase (marked in blue) and the network phase (marked in gray) shown in (**b**). The network is represented via a layer wrapped

on the pore structures, as shown in **c**, which is selected as the growing environment of those orientated carbon fibers.

Fiber insertion

Figure S6 depicts the 3D mesoscale models containing orientated fibers embedded in the network phase. As we introduced above, the 3D network skeleton (marked in gray in **Figure S5**) is firstly generated in a prescribed specimen domain, which is determined by the orientated pore structures marked in green in **Figure S5**. Then those straight fibers are placed in the network matrix with a fixed direction parallel with the X- and Z axis (**Figure S6**). The fiber model was meshed using the 3D beam elements. And the interfacial relationship between fiber and network matrix was simulated using an advanced coupling method named the Constrained_Beam_In_Solid (CBIS) algorithm in LS-DYNA. Utilizing the CBIS algorithm, the sliding and debonding behaviors of fibers could be well modeled. Additionally, a simplified two-phase linear-elastic constitutive model (Equation S16) was adopted to simulate the bond-slip interfacial behavior between fibers and scaffold matrix, such as the bonding and debonding behaviors. The details for the simplified two-phase linear-elastic model were referred to the literature [10].

$$\tau = \begin{cases} G_s S & S \leqslant S_u \\ \tau_u & S > S_u \end{cases} \tag{S16}$$

where G_s is the bond shear modulus, τ_u is the bond shear strength, and S_u is the slip shear strain.

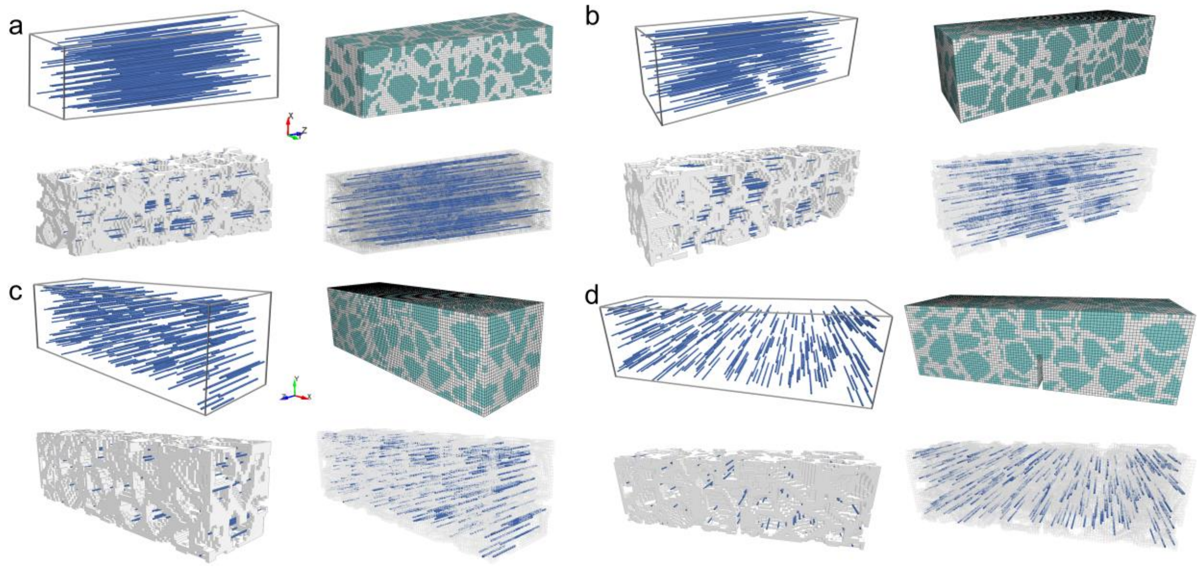

Figure S6 3D mesoscale models of the face-bridging fiber reinforced composite including straight fiber models oriented along with different directions: **a** X-axis of un-notched

specimen; **b** X-axis of notched specimen; **c** Z-axis of un-notched specimen; **d** Z-axis of notched specimen.

Finite element calculation

Following the test specimen in this work, a 3D mesoscale model with dimensions of 25 mm × 2 mm × 1.5 mm is established to perform the finite element simulation of the carbon fiber reinforced composites including orientated fibers (**Figure S7**). According to the experimental program information illustrated in the main text, a static load was uniformly applied to a steel rod that is mounted on the mid-span of the specimen. The load was controlled by displacement at a rate of 0.5 mm/min. Two steel rods were located at the bottom surface of the specimen to support it, where the distance between them was set to 24 mm. It should be noted that the frictional effect between the specimen and steel rods was not considered in this study. The *MAT_COMPOSITE_DAMAGE material model in LS-DYNA was usually selected to simulate the material behavior of resin matrix, and the details about this material model could be referred to the literature [10]. The *MAT_ELASTIC material model was selected to simulate the material behavior of carbon fiber and the network matrix, and the steel rod was assumed to be a rigid body without deformation. In accordance with the basic physical and mechanical properties, the critical model parameters of different components, such as mass density ρ, Young's modulus E, Poisson's ratio μ, tensile strength f_t, and compressive strength f_c, have been determined and listed in **Table S1**. For network matrix mechanical performance parameters, we derived them from three-point bending and compression tests (**Figure S8**). The f_t represents the bending failure strength, f_c is the compression failure strength, and E_t, E_c are calculated by dividing stress by strain from the stress-strain curves, respectively.

The above established finite element model and corresponding material models would be adopted for the numerical simulation of the composites in the ANSYS LS-DYNA software.

Table S1 Materials model parameters of different mesoscale components

Mesoscale components	Material model	Key material parameter
Resin matrix	*MAT_COMPOSITE_DAMAGE model	ρ=1.33g/ cm^3<br>E=2.0GPa<br>μ=0.37<br>f_c=80MPa<br>f_t=105MPa

Carbon fiber	*MAT_ELASTIC	ρ=1.75g/cm^3<br>E=228GPa<br>μ=0<br>f_t=3.5GPa
Network matrix with MWCNT	*MAT_ELASTIC	ρ=1.50g/ cm^3<br>E_t=2.5GPa<br>E_c= 9.8GPa<br>μ=0.38<br>f_c=90MPa<br>f_t=120MPa
Network matrix without MWCNT	*MAT_ELASTIC	ρ= 1.33g/ cm^3<br>E_t=2.0GPa<br>E_c= 9.2GPa<br>μ=0.37<br>f_c=80MPa<br>f_t=105MPa
Steel rod	*MAT_RIGID	ρ=7.80g/cm^3<br>E=210GPa<br>μ=0.3<br>f_t=210MPa

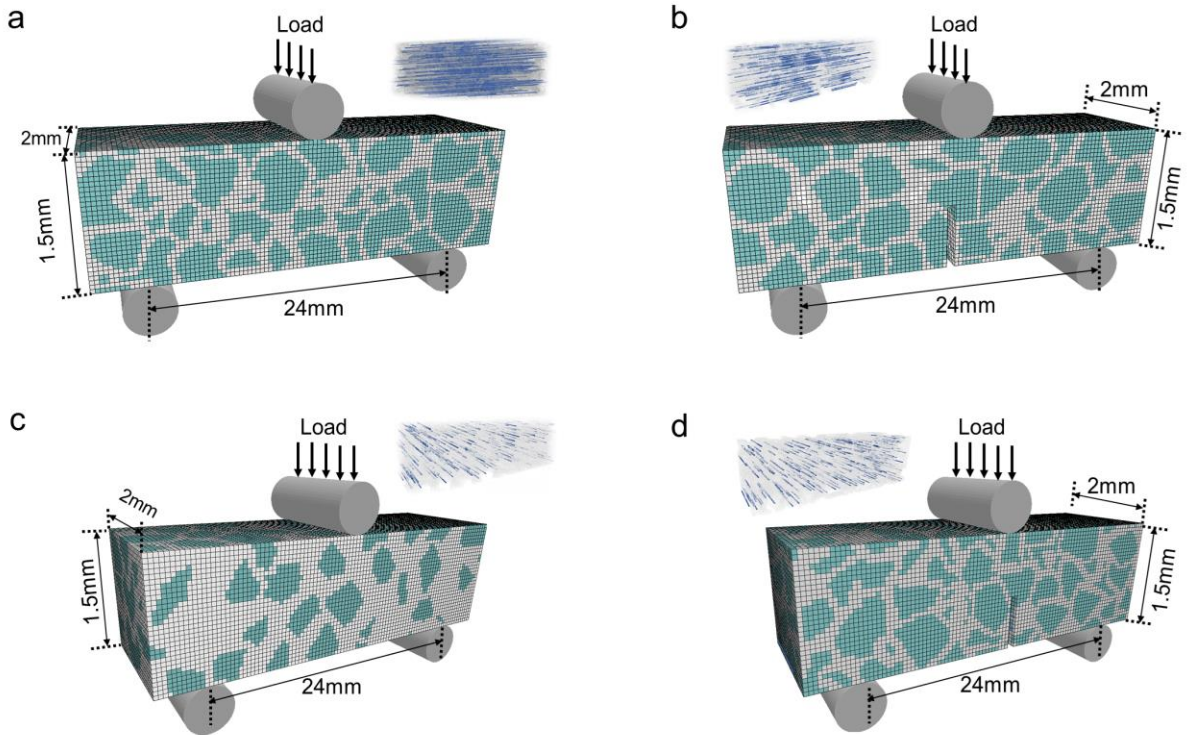

Figure S7 3D finite element model of the BFFs subjected to three-point bending load: **a** X-axis of un-notched specimen; **b** X-axis of notched specimen; **a** Z-axis of un-notched specimen; **d** Z-axis of notched specimen.

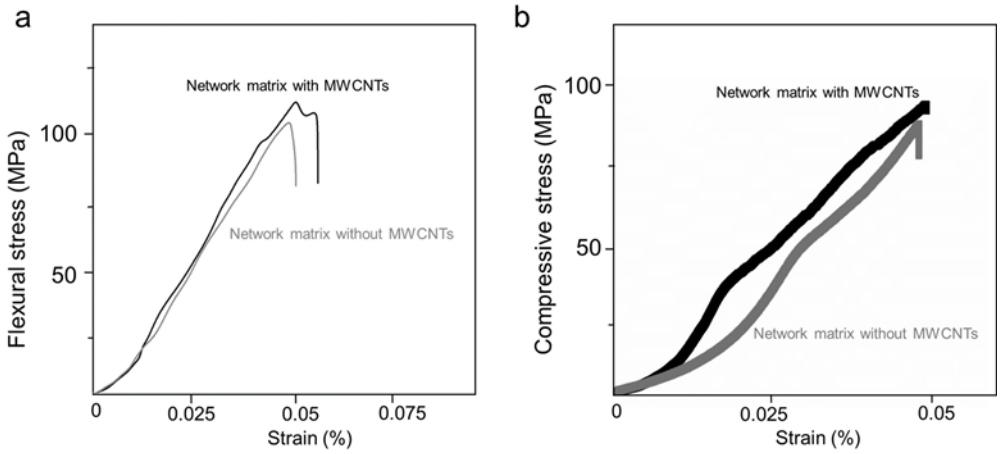

Figure S8 Mechanical properties test of the carbon network matrix: **a** three-point bending test. **b** compression test.

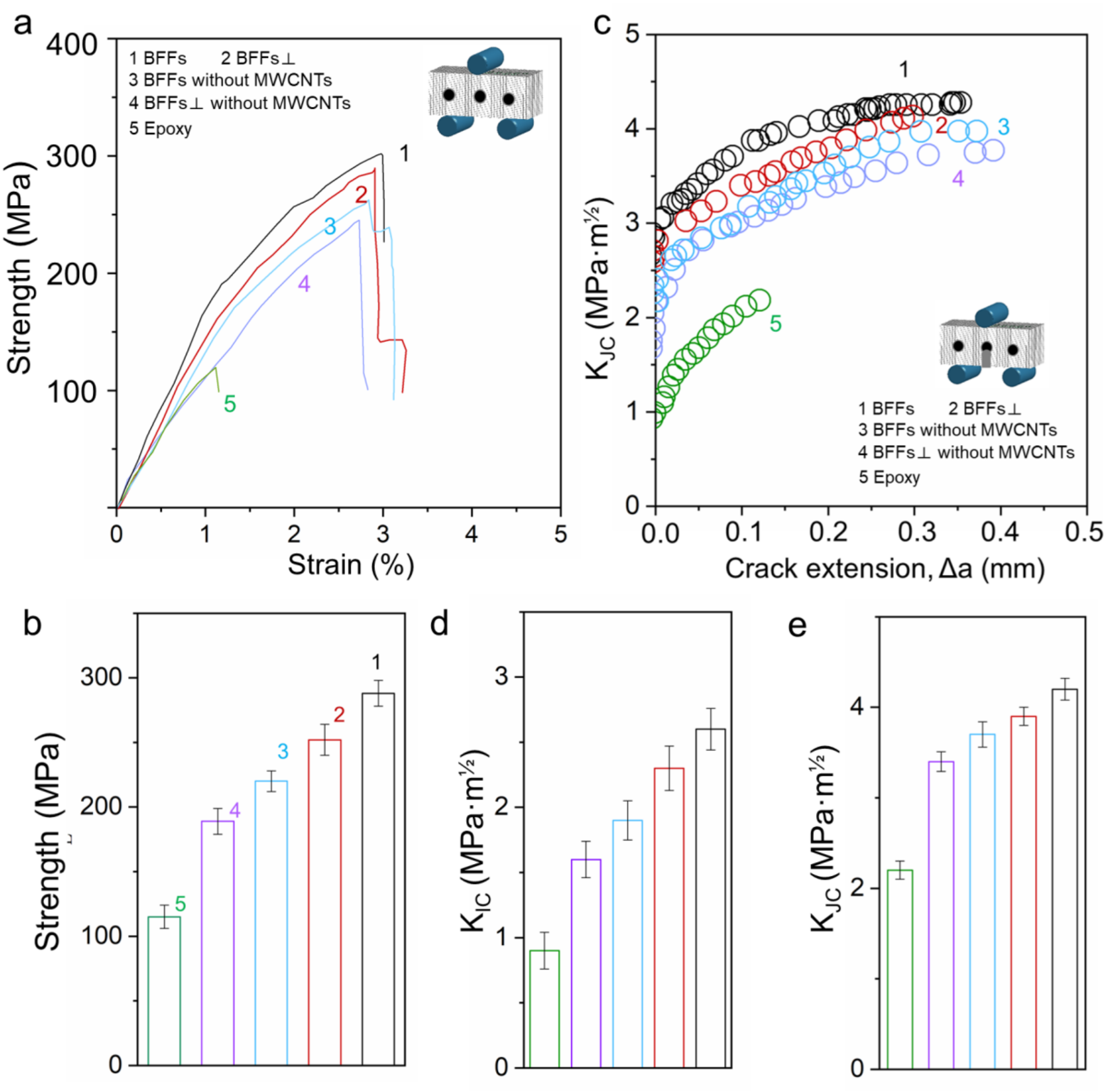

Figure S9 The mechanical properties of the BFFs loaded in the direction separating the CFs. **a** Presents typical flexural stress-strain curves and strength results (**b**), while (**c**) displays notched flexural stress-strain curves, focuses on fracture toughness, specifically crack initiation (K_{IC}) (**d**) and stable crack propagation (K_{JC}) (**e**).

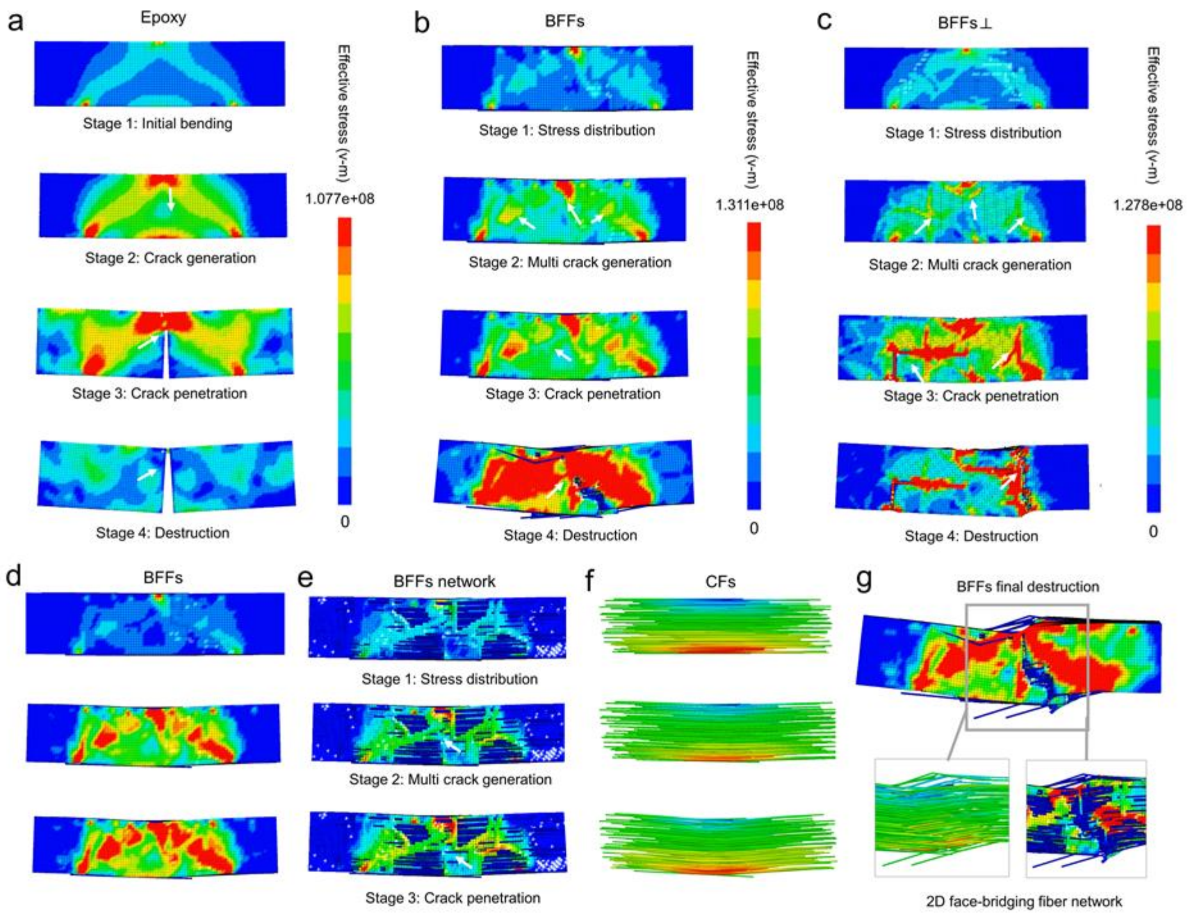

Figure S10 The fracture behavior obtained from finite element (FE) simulation: **a** Epoxy resin; **b** BFFs; **c** BFFs⊥. Stress distributions of different components in the BFFs: **d** Stress distribution in the overall BFF; **e** Stress distribution in the cellular network that bridges CFs; **f** Perspective view of the CFs. **g** Analysis of final failure surface in FE simulation.

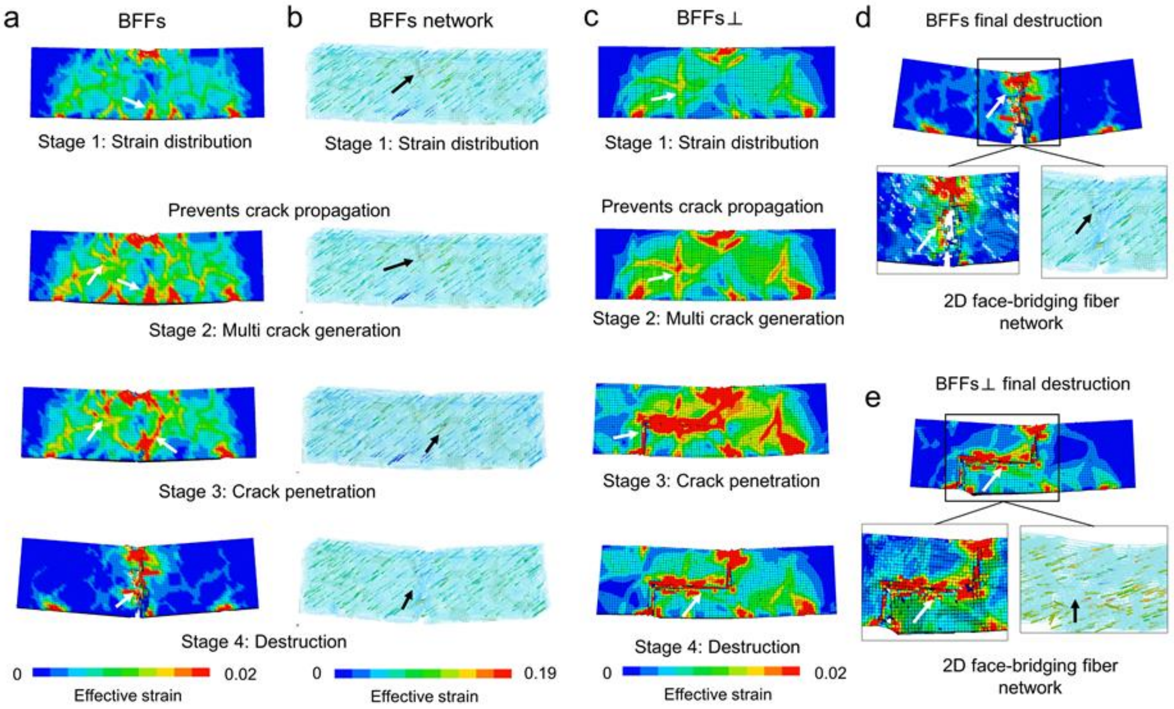

Figure S11 Three-point bending FE simulation results of failure behavior (loaded in the direction separating the CFs): The strain distributions of (**a**) the BFFs, (**b**) the BFF network, (**c**) the BFFs⊥; The final destructions of (**d**) the BFFs and (**e**) the BFFs⊥.

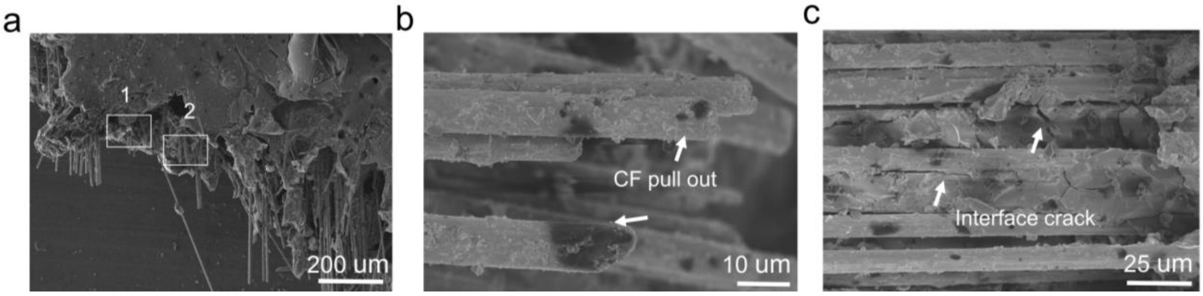

Figure S12 a SEM image of BFFs⊥ failure surface; **b** SEM image zoomed in at point 1 in (**a**); c SEM image zoomed in at point 2 in (**a**).

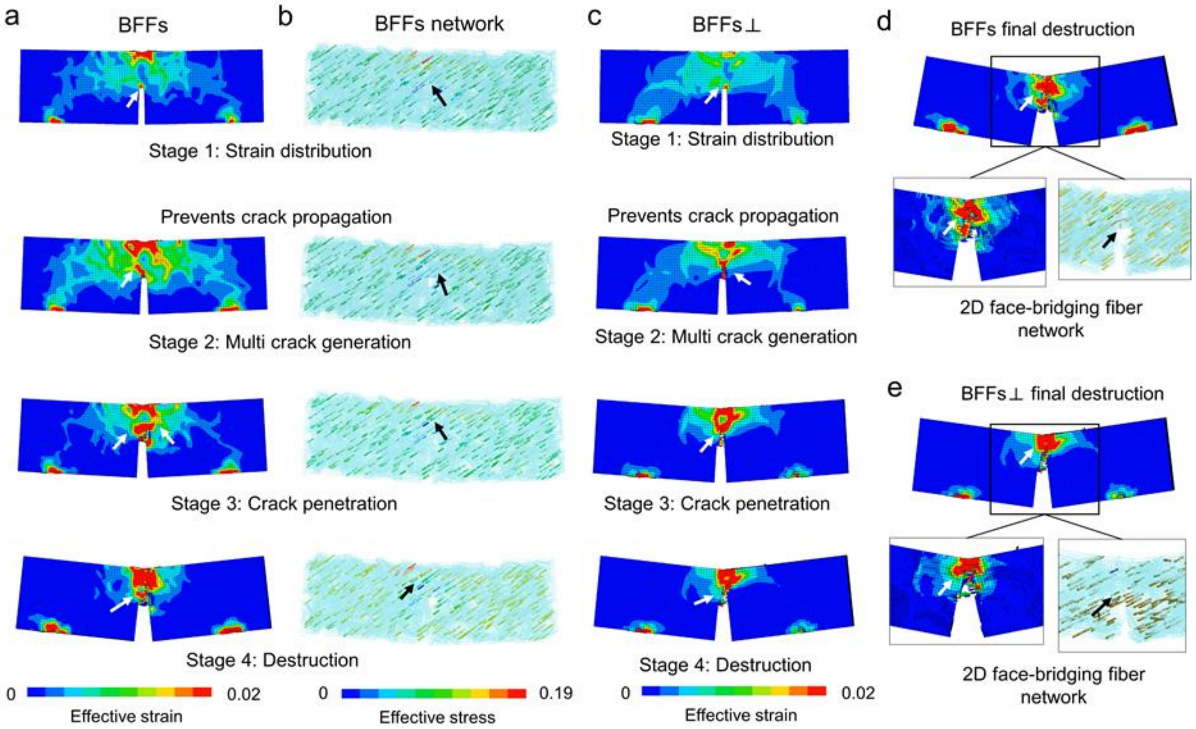

Figure S13 Notched three-point bending FE simulation results of failure behavior (loaded in the direction separating the CFs) : The strain distribution of (**a**) the BFFs, (**b**) the BFF network, and (**c**) the BFFs⊥. The final destructions of (**d**) the BFFs and (**e**) the BFFs⊥.

As illustrated in **Figure S14**, BFFs demonstrate a higher storage modulus compared to epoxy resins. This increase in storage modulus can be attributed to the rigid interface, which effectively restricts the mobility of epoxy resin molecules within the interface region, thus enhancing interfacial adhesion and stress transfer between the cellular carbon network reinforcements and the resin interface.

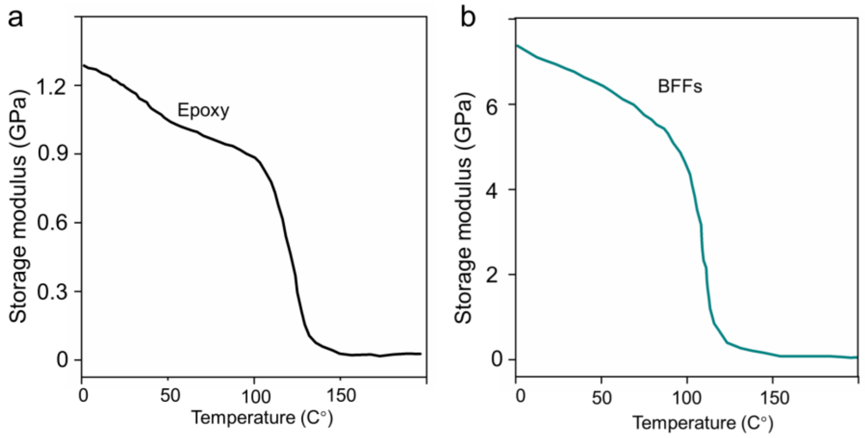

Figure S14 DMA results of **a** Epoxy, **b** BFFs.

The distribution of MWCNTs in the BFFs were imaged using a high-resolution transmission electron microscope (HRTEM, JEM-2100F, JEOL Japan), as show in the **Figure S15**. The results indicate that certain MWCNTs are randomly distributed on the surface of the carbon. Moreover, some MWCNTs are fused with the carbon and are evenly dispersed on the CF surface. Additionally, there are instances where MWCNTs act as anchors, attaching themselves to the surface of the CF and forming an interlocking structure with the resin, thus leading to an enhancement in interface performance.

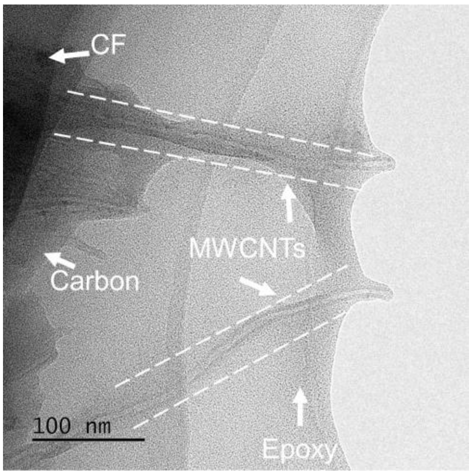

Figure S15 TEM image of the MWCNTs distribution in the BFFs.

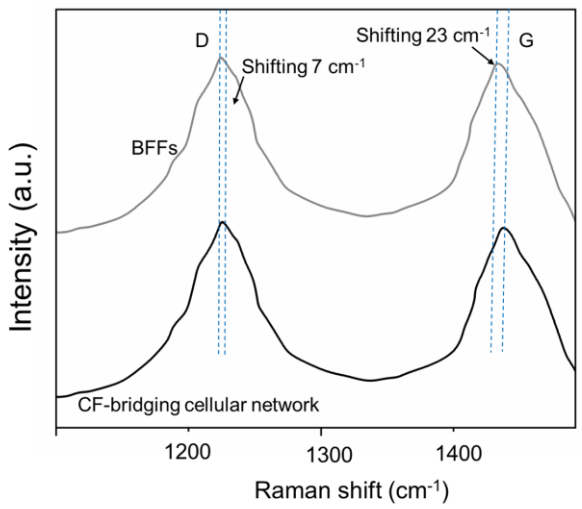

Figure S16 Raman spectra of BFFs and CF-bridging cellular network

Table S2 The mechanical properties of published epoxy composites

Materials	Density	Flexural strength (MPa)	Fracture toughness (K_{IC}) ($MPa{\cdot}m^{1/2}$)	Specific flexural strength ($MPa /g.cm^{-3}$)	References
MXene/epoxy composites	~1.25	~157	~1.07	~125.6	[11]
Lamellar rGO/epoxy	~1.24	~120	~2.9	~96.7	[12]
MTM/PVA	1.8	220	3.4	122.2	[13]
Natural AW nacre	2.64	~124	~4	46.9	[14]
Synthetic nacre	2.18	~63	2.34	~29	[14]
Graphene/epoxy	~1.3	~96	~1.5	~73.8	[15]
f-GO/epoxy	~1.25	~155	~0.7	~124	[16]
GA/epoxy	~1.3	~93	~1.5	~71.5	[17]
Al_2O_3/epoxy	~2.18	~185	~2.6	~85.0	[18]
Conductive nacre	1.24	~164	~4.86	132.3	[19]
3D Interconnected Graphene Foam/Epoxy Composites	~1.2	~128	~1.78	~106.7	[20]
Epoxy-silanized chopped carbon fibers	1.21	~58	~1.09	~47.9	[21]
Epoxy-melamine functionalized carbon nanotubes	N/A	105	2.02	N/A	[22]
Epoxy-bisphenol-A functionalized graphene oxide	N/A	~94.8	~0.669	N/A	[23]
Epoxy Composite Based on Non-oxidized Graphene Flake Aerogel	N/A	~100	~1.74	N/A	[24]
Carbon fiber epoxy composites	N/A	~1000	~2.44	N/A	[25]
Natural bamboo	0.94	~148	~7.822.31	~157.4	[26, 27]
Our work BFFs	1.27	~430	~5.4	~338.6	

PMMA: polymethyl methacrylate, rGO: reduced graphene oxide, MTM: Montmorillonite, AW: anodonta woodiana, f-GO: functionalized graphene oxide, PVA: polyvinyl alcohol, GA: amine-terminated poly(butadieneacrylonitrile)-modified graphene oxide.

Table S3 The mechanical properties of natural bamboo

Material	Tensile strength (MPa)	Modulus (GPa)	Flexural strength (MPa)	Density (g/cm^3)	Work of fracture ($MJ\ m^{-3}$)	Reference
Natural Bamboo	298	19.2	148	9.8	2.31	[27]

Table S4 Summary of improvements in strengthening and toughening of carbon reinforced epoxy resin matrix with different architecture types

Architecture types	Reinforcement	Preparation method	Maximum enhancement of strength (%)	Maximum increments of toughness (%)	References
Random	DWCNTs	Amino functionalization + shear mixing + curing	8.4	43.1	[28]
Random	MWCNTs	Ozone treatment + shear mixing + three-roll milling + curing	22.6	52.2	[29]
Random	RGO	Solution mixing + ball milling + curing	8.6	52	[30]
Random	GO	Solution mixing + evaporation + three-roll milling + curing	13.8	62.7	[31]
Random	GNP	Solution mixing + evaporation + curing	21.4	167.9	[32]
Laminate	SWCNTs	Slide coating + LBL	93.5	148.8	[33]
Network	CNT Sponge	CVD +	64	250	[34]

		infiltration			
Network	Graphene aerogel	Freeze-casting	11	69	[35]
Network	Graphene foam	Freeze-drying	67	132	[36]
Alignment	Graphene aerogel	Directional freeze-casting	2	64	[15]
Alignment	Graphene flake aerogel	Directional freeze-casting	10	76	[24]
Alignment	Graphene aerogel	Directional freeze-casting	14	320	[12]
Alignment	Graphene aerogel	Directional freeze-casting	18	261	[37]
Our work	Bamboo-inspired structure of cell face-bridging fibers	Freeze-casting & drying + carbonization + resin infiltration	290	440	

GNP: graphene platelet

References

[1] S.W. Sihn, R.Y. Kim, K. Kawabe, S.W. Tsai. Experimental studies of thin-ply laminated composites. Compos. Sci. &Technol. 67, 6, 996–1008 (2007).

[2] H.W. Zhao, et al. Multiscale engineered artificial tooth enamel. Science, 375,551 556 (2022).

[3] H. Wang, K. Jin, C. Wang, X.Z. Guo, Z. Chen, J. Tao. Effect of fiber surface functionalization on shear behavior at carbon fiber/epoxy interface through molecular dynamics analysis. Compos. Part A: Appl. Sci. & Manuf., 126, 105611 (2019).

[4] H. Wang, K. Jin , J. Tao. Improving the interfacial shear strength of carbon fibre and epoxy via mechanical interlocking effect. Compos. Sci. &Technol. 200, 108423 (2020).

[5] Y.L. Li, S.J. Wang, Q. Wang. A molecular dynamics simulation study on enhancement of mechanical and tribological properties of polymer composites by introduction of graphene. Carbon, 111, 538–545 (2017).

[6] H. Sun. COMPASS: an ab initio force-field optimized for condensed-phase applications overview with details on alkane and benzene compounds. J. Phys. Chem. B., 102 (38), 7338–7364 (1998).

[7] J.M.D. Sousa, A.L. Aguiar, E.C. Girão, A.F. Fonseca, A.G. Souza Filho, D.S. Galvão. Computational study of elastic, structural stability and dynamics properties of penta-graphene membrane. Chem. Phys., 542, 111052 (2021).

[8] H. Watanabea. Stability of velocity-Verlet- and Liouville-operator-derived algorithms to integrate non-Hamiltonian systems. Chem. Phys., 149, 154101(2018).

[9] D.J. Evans, B.L. Holian. The nose–hoover thermostat. J. Chem. Phys., 83 (8), 4069–4074 (1985).

[10] L.S.T.C. LS-DYNA version 971 keyword user's manual. Livermore Software Technology Corporation, California, USA (2007).

[11] L. Liu, G.B. Ying, D. Wen, et al. Aqueous solution-processed MXene (Ti3C2Tx) for non-hydrophilic epoxy resin-based composites with enhanced mechanical and physical properties. Mater. & Des., 197, 109276 (2021).

[12] C. J. Huang, J. S. Peng, S. J. Wan, A. P. Tomsia, L. Jiang, Q. F. Cheng. Ultra-Tough Inverse Artificial Nacre Based on Epoxy-Graphene by Freeze-Casting. Angew. Chem., 131,7718–7722 (2019).

[13] M. Morits, T. Verho, J. Sorvari, V. Liljeström, et al. Toughness and Fracture Properties in Nacre-Mimetic Clay/Polymer Nanocomposites. Adv. Funct. Mater., 27, 1605 (2017).

[14] L.B. Mao, et al. Synthetic nacre by predesigned matrix-directed mineralization. Science, 354,107–110 (2016).

[15] Z.Y. Wang, X. Shen, M.A. Garakani, X.Y. Lin, et al. Graphene Aerogel/Epoxy Composites with Exceptional Anisotropic Structure and Properties. ACS Appl. Mater. & Inter., 7 (9), 5538–5549 (2015).

[16] Y.J. Wan, L.X. Gong, L.C. Tang, L.B. Wu, J.X. Jiang. Mechanical properties of epoxy composites filled with silane-functionalized graphene oxide. Compos. Part A: Appl. Sci. & Manuf., 64, 79–89 (2014).

[17] Y.T. Park, Y.Q. Qian, C. Chan, T Suh. Epoxy Toughening with Low Graphene Loading. Adv. Funct. Mater., 25, 575–585 (2015).

[18] R. Libanori, R.M. Erb, A.R. Studart. Mechanics of Platelet-Reinforced Composites Assembled Using Mechanical and Magnetic Stimuli. ACS Appl. Mater. & Inter. 5 (21), 10794–10805 (2013).
[19] H.G. Wang, R.J. Lu, J. Yan, J.S. Pen. Tough and Conductive Nacre-inspired MXene/Epoxy Layered Bulk Nanocomposites. Angew. Chem. Int. Ed., 62, e202216874 (2023).
[20] J.J. Jia, X.Y. Sun, X.Y. Lin, X. Shen, Y.W. Mai, J.K. Kim. Exceptional Electrical Conductivity and Fracture Resistance of 3D Interconnected Graphene Foam/Epoxy Composites. ACS Nano, 8 (6), 5774–5783 (2014).
[21] S. Das, S. Halder, J. Wang, M.S. Goyat, A.A. Kumar, Y. Fang. Amending the thermo-mechanical response and mechanical properties of epoxy composites with silanized chopped carbon fibers. Compos. Part A: Appl. Sci. & Manuf., 102, 347–356 (2017).
[22] J.M. Cha, G.H. Jun, J.K. Park, J.C. Kim, H.J. Ryu, S.H. Hong. Improvement of modulus, strength and fracture toughness of CNT/Epoxy nanocomposites through the functionalization of carbon nanotubes. Compos. Part B: Eng., 129, 169–179 (2017).
[23] Y.J. Wan, L.C. Tang, L.X. Gong, D. Yan, et al. Grafting of epoxy chains onto graphene oxide for epoxy composites with improved mechanical and thermal properties. Carbon, 69, 467–480 (2014).
[24] J. Kim, N.M. Han, J.M. Kim, J.H. Lee, J.K. Kim, S. Jeon. Highly Conductive and Fracture-Resistant Epoxy Composite Based on Non-oxidized Graphene Flake Aerogel. ACS Appl. Mater. & Inter. 10 (43), 37507–37516 (2018).
[25] S.M. Cai, Y.Li, H.Y. Liu, Y.W. Mai. Effect of electrospun polysulfone/cellulose nanocrystals interleaves on the interlaminar fracture toughness of carbon fiber/epoxy composites. Compos. Sci.&Technol.,181, 107673 (2019).
[26] Z. Ba, H. Luo, J. Guan, et al. Robust flexural performance and fracture behavior of TiO2 decorated densified bamboo as sustainable structural materials. Nat. Commun. 14, 1234 (2023).
[27] Z. H. Li, C.J. Chen, R.Y. Mi, W.T. Gan, et al. A Strong, Tough, and Scalable Structural Material from Fast-Growing Bamboo. Adv. Mater., 32, 10, 1906308 (2020).
[28] F. Gojny, M. Wichmann, B. Fiedler, K. Schulte. Influence of different carbon nanotubes on the mechanical properties of epoxy matrix composites – a comparative study. Compos. Sci. &Technol., 65:2300–13 (2005).
[29] L. Tang, H. Zhang, J. Han, X. Wu, Z. Zhang. Fracture mechanisms of epoxy filled with ozone functionalized multi-wall carbon nanotubes. Compos. Sci. &Technol., 72, 7–13 (2011).
[30] L. Tang, Y. Wan, D. Yan, Y. Pei, L. Zhao, Y. Li, et al. The effect of graphene dispersion on the mechanical properties of graphene/epoxy composites. Carbon, 60, 16–27 (2013).
[31] D.R. Bortz, E.G. Heras, I. Martin-Gullon. Impressive fatigue life and fracture toughness improvements in graphene oxide/epoxy composites. Macromolecules, 45, 238–245 (2011).
[32] S. Han, Q. Meng, S. Araby, T. Liu, M. Demiral. Mechanical and electrical properties of graphene and carbon nanotube reinforced epoxy adhesives: experimental and numerical analysis. Compos. Part A: Appl. Sci. & Manuf., 120, 116–126 (2019).
[33] C. Sui, Z. Pan, R.J. Headrick, Y. Yang, C. Wang, J. Yuan, et al. Aligned-SWCNT film laminated nanocomposites: role of the film on mechanical and electrical properties. Carbon, 139, 680–687(2018).

[34] Y. Chen, H. Zhang, Y. Yang, M. Wang, A. Cao, Z. Yu. High-performance epoxy nanocomposites reinforced with three-dimensional carbon nanotube sponge for electromagnetic interference shielding. Adv. Funct. Mater., 26, 447–455 (2016).
[35] N.M. Han, Z.Y. Wang, X. Shen, Y. Wu, et al. Graphene Size-Dependent Multifunctional Properties of Unidirectional Graphene Aerogel/Epoxy Nanocomposites. ACS Appl. Mater. & Inter., 10 (7), 6580–6592 (2018).
[36] K. Wang, W. Wang, H. Wang, L. Liu, Z. Xu, H. Fu, L. Zhao, X. Zhang, L. Chen, Y. Zhao. 3D graphene foams/epoxy composites with double-sided binder polyaniline interlayers for maintaining excellent electrical conductivities and mechanical properties. Compos. Part A: Appl. Sci. & Manuf., 110, 246 (2018).
[37] J. S. Peng, C. J. Huang, C. Cao, E. Saiz, Y. Du, et al. Inverse nacre-like epoxy-graphene layered nanocomposites with integration of high toughness and self-monitoring. Matter, 2, 220 (2020).
